# Importance of Medico-Dental Education

**Published:** 1847-10

**Authors:** 


*Importance of Medico-dental Education.*—We published in the fourth number of the last volume of the Journal an ably written article from our London correspondent, Dr. J. Robinson, on the importance and value of a knowledge of the collateral branches of medicine and surgery in connection with dentistry, and another article upon the same subject from the same writer will be found in the present number. The value of such knowledge to the dental surgeon is most conclusively shown, as all must admit after perusing these ably written papers. The subject will be continued in the next number, and we bespeak for the series an attentive reading.—*Balt. Ed.*

